# Welfare and Performance of Finishing Pigs Kept at Two Group Sizes on Ad Libitum vs. Restricted Feeding

**DOI:** 10.3390/ani16091342

**Published:** 2026-04-28

**Authors:** Inger Lise Andersen, Oda Braar Wæge, Marko Ocepek, Signe Lovise Thingnes, Kristine Hov Martinsen, Anne Stine Ekker, Ruth C. Newberry

**Affiliations:** 1 Department of Animal and Aquacultural Sciences, Faculty of Biosciences, Norwegian University of Life Sciences, P. O. Box 5003, 1432 Ås, Norway; 2 Norsvin S. A., Storhamargata 44, 2317 Hamar, Norway; signe-lovise.thingnes@norsvin.no (S.L.T.);; 3 Felleskjøpet Fôrutvikling, Nedre Ila 20, 7018 Trondheim, Norway

**Keywords:** growing–finishing pigs, bite marks, human approach test, animal welfare, space availability, feed availability, locomotion disorders

## Abstract

This 2 × 2 factorial study examined the welfare and performance of finishing pigs at two group sizes (9 or 18 pigs) over 12 weeks. For each set of groups of either 9 or 18 pigs, half of the pigs in each group size were fed ad libitum, while the others received a mildly restricted ration. Treatments were assigned to 16 partially slatted floor pens in a randomized block design, with a floor space of 1.15 m^2^/pig. Except in Week 1, there were proportionally fewer pigs with ear (*p* = 0.020) and tail (*p* < 0.0001) bite marks in groups of 18 than in groups of 9. Ear bite marks declined over time in both group sizes (*p* < 0.0001). There was also a significant interaction between group size and week regarding severe bite marks on the ears (*p* < 0.0002). Tail bite mark prevalence increased over time in the smaller groups but decreased in the larger groups (interaction: *p* < 0.001). A higher proportion of pigs in smaller groups sought human contact in Weeks 1 and 6, but this measurement equalized by Week 10 (interaction: *p* = 0.008). There were proportionally more pigs with tucked tails in the smaller groups in Week 1 but not in later weeks (interaction: *p* < 0.0001). Group size did not influence pig cleanliness or locomotion disorders. Ad libitum (vs. restricted) feeding increased average daily gain (*p* < 0.001), feed intake (*p* = 0.002), and slaughter weight (*p* = 0.030). Results suggest better welfare in the larger than in the smaller groups.

## Simple Summary

Some commercial farmers of growing–finishing pigs want to merge two adjacent pens to achieve larger groups of pigs. The aim of this research was to compare the welfare and performance of finishing pigs housed in two group sizes (9 or 18 pigs). Half the groups were fed ad libitum, and half receiving restricted feed three times daily. All groups were provided with a usable floor space of 1.15 m^2^/pig. Welfare indicators assessed three times during the study showed that a lower proportion of pigs had ear and tail bite marks in groups of 18 compared to 9 pigs. Ear bite mark prevalence declined over time, and more pronouncedly at the larger group size. Tail bite mark prevalence increased over time in the smaller groups but decreased in the larger groups. There was a higher proportion of pigs with tucked tails in the smaller groups, while the proportions with curled and wagging tails were unaffected by group size. Group size did not influence pig cleanliness or locomotion disorders. Feeding system had no impact on the welfare indicators, but ad libitum feeding improved growth and slaughter weight. In conclusion, the results support doubling the group size of small groups of finishing pigs by merging neighbouring pens.

## 1. Introduction

In commercial production, it may be desirable to double the group size of growing–finishing pigs (otherwise known as fattening pigs or, in the final feeding phase prior to slaughter, finishing pigs). This can be done by removing the pen divider between two adjacent small pens, thereby maintaining the same stocking density. Doubling the group size is simple if the pens are comprised of removeable panels, while incurring a modest building cost if the pen walls are permanently fixed. This gives pigs a larger living space in which to move around and interact with a greater diversity of individuals, which may increase mental stimulation, reduce the risk of developing locomotion disorders and tail biting behaviour, and improve ventilation. It could, thus, improve pig welfare and performance if such progress is not constrained by behaviour problems such as fighting, bullying and poor dunging patterns.

Studies have shown that, at the same stocking density, aggression levels among growing–finishing pigs can vary depending on group size. It has been documented that pigs in small groups of 6–12 pigs tended to exhibit more aggression per pig compared to pigs in larger groups of 24 pigs, with a higher proportion of individuals engaging in fights [1]. Similar findings have been reported in other studies [2,3,4], although some research suggests that aggression levels can remain relatively constant as group size increases [5]. A recent Norwegian field survey of commercial finishing pigs in groups ranging from 2 to 201 pigs revealed that larger groups had proportionally fewer individuals with bite marks on their ears and body [6]. Bite marks on the ears and body typically result from social aggression among unfamiliar pigs when mixed [7]. However, tail biting, a behaviour linked to redirected foraging and stress in high-density environments, is not a form of social aggression [8,9,10]. No clear association between group size and tail biting was detected in studies on pigs with docked tails [11,12] or in the recent Norwegian epidemiological survey of pigs with intact tails [6]. Nevertheless, controlled experiments on the impact of group size on tail biting in pigs with intact tails are lacking.

In addition to effects on aggression, group size has been reported to influence feeding behaviour. It has been observed that pigs housed in groups of 20, compared to those in smaller groups of 5, 10, or 15, made fewer but longer visits to the feeder, consuming more feed per visit and eating at a faster pace [2]. Such changes in feeding strategy with increasing group size may be associated with differences in performance. There are reports of associations between group size and performance measures, such as slightly reduced weight gain [6] or higher feed consumption to maintain a similar weight gain [13]. However, other studies found no difference in growth performance across different group sizes when pigs were kept in relatively small groups in indoor pens [2,14]. Further research is needed to resolve these inconsistencies.

Growing–finishing pigs are typically fed ad libitum on dry concentrates from automated feed dispensers. Some degree of feed restriction may increase feed efficiency but also alter the feeding pattern. For example, one study found that mild feed restriction resulted in decreased feed intake, fewer visits to the feeder, and less time feeding but increased consumption per visit [15]. Whether group size affects how pigs respond to feed restriction is unclear, but it could potentially affect both performance and welfare.

The present study selected a few core welfare indicators that were expected to be sensitive to the treatments and within the goal of researchers not being required to spend more than 2 h at the farm to assess all finishers. In the current study, we compared groups of 9 pigs (a traditional small group size for finishing pigs) vs. groups of 18 pigs (kept in double-sized pens formed by removing the dividing wall between adjacent pairs of pens). The study extended from pig delivery at approximately 10 weeks of age until slaughter at approximately 22 weeks of age. Our objective was to assess the welfare and performance of finishing pigs when housed at these two group sizes in partially slatted pens at the same stocking density under ad libitum vs. mildly restricted feed conditions. Because resources such as specific feeding and lying locations are less easily defended by specific individuals in larger groups [1,16,17], we hypothesized that the prevalence of bite marks would be lower in larger groups. We hypothesized that feed restriction would increase competition and thereby result in a higher prevalence of bite marks. Therefore, we predicted that the proportion of pigs with bite marks on the ears, body and tail would be lower in the larger groups, especially when fed ad libitum. Furthermore, we predicted that we would see a higher proportion of pigs with curled and wagging tails (indicating positive affective states [18]) as opposed to tails tucked between the legs (associated with tail biting [19]). We also questioned whether pig cleanliness, locomotion disorders and confidence in approaching unfamiliar people would be affected by group size. Regarding performance, we predicted that restricted feeding would result in higher feed efficiency but reduced growth due to lower feed intake compared to the results for ad libitum feeding. Finally, we questioned how the measured variables would change over time during the finishing period.

## 2. Materials and Methods

### 2.1. Ethics Declaration

All procedures were conducted in accordance with national legislation (www.lovdata.no) and institutional/university standards. The study did not involve any specific handling of the pigs or disturbance of the farmers’ daily routines. The pigs were mixed upon arrival at the farm (on Day 1), which is part of the regular management routine.

Only non-invasive methods were used. The pigs were visually observed in their home pens, with limited human interaction, initiated only if the pigs themselves approached. None of the procedures in this study were expected to cause harm or distress to the animals. Animal care and use followed normal commercial practices in Norwegian pig production. The housing setup was agreed upon by the farmer, who wished to evaluate whether removing walls to increase group size could be done without adding tional building costs. Felleskjøpet Agri S.A., Oslo, NO, had a written contract with the farmer stating that he was willing to host experimental work that could benefit Norwegian pig production.

### 2.2. Pigs, Housing, Feeding and Management

The experiment was conducted at a commercial farm in Øyer, Norway. This farm is often used for feed trials by a Norwegian feeding company, and the pen design is representative of Norwegian farms with small-group housing. DDZL Duroc x TN70 pigs (Norsvin S.A., Hamar, Norway) were delivered at approximately 10 weeks of age. Prior to delivery, they were reared in litter groups in crate-free farrowing pens until weaning and mixing at 5 weeks of age. Males were castrated at 1 week of age with the use of anaesthetic and analgesic, while teeth and tails were left intact. Upon arrival at the grower–finisher facility, they were housed in solid-walled pens with partially slatted concrete floors (1/3 slatted, 2/3 solid) coated with anti-slip epoxy resin. The room temperature was 20 to 22 °C upon arrival and was gradually decreased to 16 to 17 °C by the end of the finishing period. Windows along the south wall provided natural light, which was supplemented by LED lighting from 07:00–16:00 h.

The same pelleted dry feed (Felleskjøpet Agri S.A., Oslo, Norway), with 9.7 MJ NE/kg and 0.87 g SID Lysine/MJ [20], was fed throughout the experimental period, with barley, oats, rapeseed cake, and sunflower meal as the main ingredients. The pigs had free access to water from nipple drinkers. The pigs were given sawdust as a bedding material in the solid-floor resting area, and rooting material was added twice a day. This consisted of a fistful of hay per every nine pigs in the morning. In the evening, they either got the same amount of hay, a few pages of newspaper, wood chips, or large high-fibre pellets with 80% beet pulp (Format Trivsel, Felleskjøpet Agri S.A., Oslo, Norway). Additional environmental enrichment was supplied in the form of small hanging rubber tyres. In all pens, the floor space per pig was 1.15 m2, and access to resting areas, drinking nipples, enrichment materials, rubber tyres, and slatted dunging areas were all standardized on a per pig basis. All pigs were monitored twice daily for health and weighed weekly. No mortality or disease outbreaks occurred during the experimental period. The pigs were sent for slaughter after 12 weeks.

### 2.3. Experimental Design

**Figure 1:**
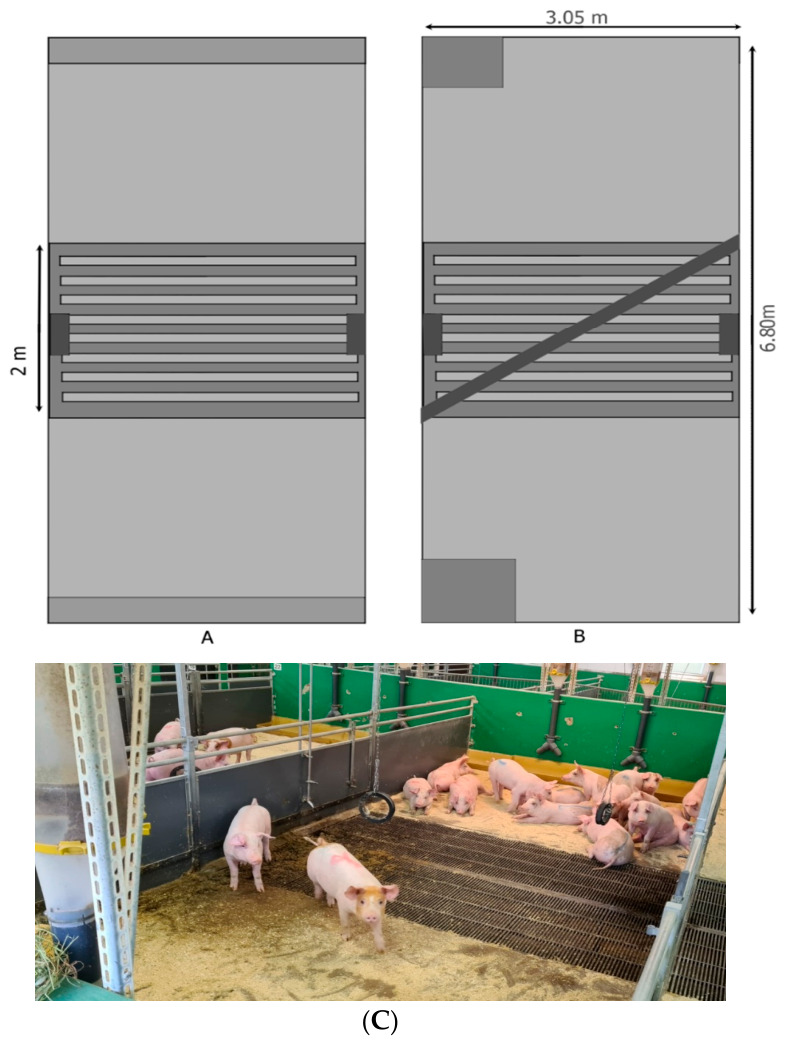
(**A**) A large pen (without pen partition) housing 18 pigs with two water nipples (dark grey rectangle on each side of the central slatted area), and two hanging tyres (shown in (**C**)). The parallel dark grey lines depict the slatted floor area. The light grey areas without slats are resting areas with a solid, bedded floor. (**B**) Two small pens housing nine pigs each, divided by a solid partition placed diagonally across the slatted area, with each pen containing one nipple drinker and one hanging rubber tyre over the slats. A feed trough (dark grey) was located on the end wall of each small pen or at each end of large pens (long and shallow for restricted feeding (shown in (**A**)) or short and deep for ad libitum feeding (shown in (**B**)). All pens provided 1.15 m^2^ floor space per pig, and resting space per pig was the same for both group sizes. (**C**) Picture of a large pen with long, shallow troughs for restricted feeding three times a day.

The pigs were housed at one of two group sizes, 9 pigs (*n* = 8 pens) or 18 pigs (*n* = 8 pens), on one of two feed systems, ad libitum or restricted. For groups of 9 pigs, the pens were divided by a diagonal wall across the 2 m wide slatted floor (the standard configuration on this farm), while for groups of 18 pigs, the diagonal wall was removed, resulting in pens measuring 3.05 m by 6.80 m (Figure 1). The pens were arranged in a randomized complete block design (*n* = 4 blocks), with each block comprising four pens, one for each of the four treatment combinations. An equal number of females and castrated males were randomly assigned to each block such that half of the groups of 9 pigs had four females and five males, and the other half had five females and four males, while the groups of 18 had nine pigs of each sex.

The groups of 9 pigs had a single feeder and the groups of 18 pigs had two feeders (Figure 1). Groups on the ad libitum feeding system had a continuous supply of feed available in tall feed hoppers with short troughs (60 cm long, with a central divider) allowing two pigs to feed from the same feeder at the same time (6.7 cm feeder space/pig). Groups on the restricted feeding system received the feed in long, shallow troughs (3.05 m long), allowing all pigs to eat simultaneously (33.9 cm feeder space/pig). Their feed (average 2.8 kg/pig/day) was distributed automatically three times daily (in equal amounts at approximately 07:30, 11:30, and 15:30 h). The amount was adjusted weekly and was calculated to result in a mild (5%) restriction in feed intake compared to ad libitum feeding. 

### 2.4. Data Collection

Based on a request from the Norwegian Food Safety Authority, we designed a simplified, practical welfare assessment tool that was considered time-effective compared to extensive welfare assessment protocols such as the Welfare Quality evaluation. The assessment protocol was designed to be relevant for use by government inspectors, farmers, and pig industry advisors on Norwegian farms, enabling completion within 2.0 h per farm. Using this protocol (Table 1), welfare assessments of the pigs in each group were conducted by three trained researchers in Weeks 1 (3–4 days after mixing), 6, and 10 of the study. Each assessor collected data from a different set of pens, balanced over weeks.

In each pen, we first conducted a human approach test, followed by scoring of all pigs for tail posture category, cleanliness, locomotion disorders, and bite marks (Table 1). In the human approach test (HAT), an assessor unfamiliar to the pigs opened the pen door and moved slowly inside the pen and closed the door behind her before standing for 30 s facing the pigs with arms downwards in a relaxed position. After the 30 s, the assessor registered the number of pigs that had sought physical contact during the 30 s observation. This was followed by assessing the number with each tail posture and the number that were relatively clean. We wanted to assess tails in connection with this test because tail state may also say something about what expectations (positive or negative) the pigs have in regard to an approaching human. The point was to measure an immediate response, not that noted after the individual walked around in the pen. For locomotion disorders, as well as ear, body, and tail bite marks, the numbers of pigs per pen in the mild and severe categories were recorded and then summed to obtain overall prevalences. To ensure high inter-assessor agreement, the team members practised the method together before commencing data collection (average Pearson correlation between pairs of assessors for bite mark scores: Rh0 = 0.8). This value was achieved between two assessors regarding bite marks on the first visit, when two persons independently observed the same pigs. The result was 0.8 for all three bite mark indicators, most likely because, prior to the study, we practiced the methods during a pilot test at our university experimental farm. The rest of the indicators were pretrained and interpreted together in the pilot.

The performance variables were body weight at the start and end of the finishing period, average daily gain over the entire finishing period, average daily feed intake for the entire period, feed conversion rate, and the number of days from delivery to the farm until slaughter. From the abattoir, we obtained slaughter weight and % lean meat figures. In Norway, the slaughter weight represents the weight of the carcass after the head, legs and innards are removed, and it is estimated to be approximately 68% of the live weight (Norwegian standard).

### 2.5. Statistical Analysis

The proportion of pigs in each condition was analysed using a generalized linear mixed model (GLMM) implemented with the GLIMMIX procedure in SAS (SAS Institute Inc., Cary, NC, USA). The response variable was specified as the number of pigs in each condition out of the total number of pigs in each pen, assuming a binomial distribution with a logit link function. Fixed effects included group size (9 vs. 18), week (1, 6 and 10), feeding system (ad libitum vs. restricted), and the interactions between group size and week and between group size and feeding system. The pen was included as a random effect to account for repeated observations within the same pen. Model parameters were estimated using Laplace approximation to the likelihood. SAS uses approximation methods to decide how much independent information is available for testing each effect. We had 48 observations, resulting in a fairly complex model with three categorical predictors (group size, time, feeding system), interactions (group size × time, group size × feeding system), and a random effect: pen (16 levels). This means that the model estimates many parameters and that the random effect and class structure reduce the “independent” information.

For performance variables, mean ± SE per pen was calculated based on the number of days in the experiment for each pig. Pen means for the performance variables were analysed using a general linear model (GLM procedure in SAS 9.4), with group size, feeding system, and their interaction as fixed effects. Pen mean start weight was included as a covariate for all variables except for lean meat.

Differences were considered significant at *p* < 0.05. The least square means method with Tukey adjustment was used to detect significant pairwise differences between means. With only four replicates (pens) per treatment combination, the study is underpowered for detecting moderate effect sizes, and thus, conclusions should be drawn with caution. 

## 3. Results

### 3.1. Welfare Indicators

#### 3.1.1. Human Approach Test (HAT) and Tail Posture

**Figure 2:**
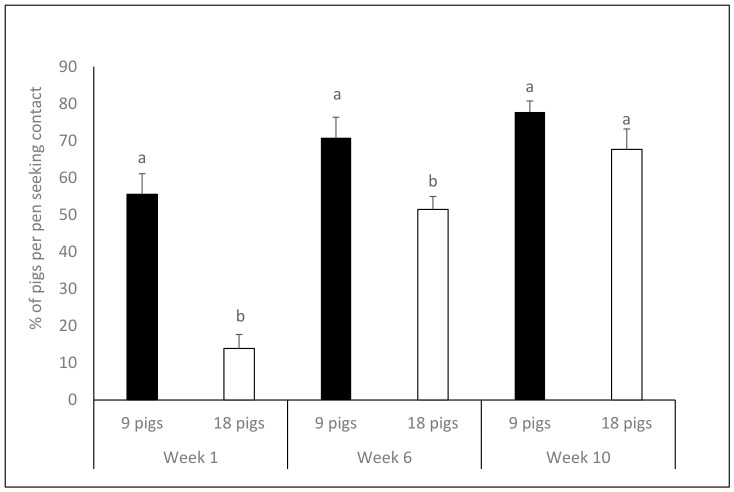
Mean + SE% of pigs per pen seeking contact with an unfamiliar human in the human approach test. Different superscripts (a, b; *p* < 0.05) denote significant differences between groups of 9 (black bars) vs. 18 (white bars) pigs within weeks (least square means method with Tukey adjustment). There is a significant difference between groups of 9 and 18 pigs in Week 1 and 6 but not in Week 10.

**Figure 3:**
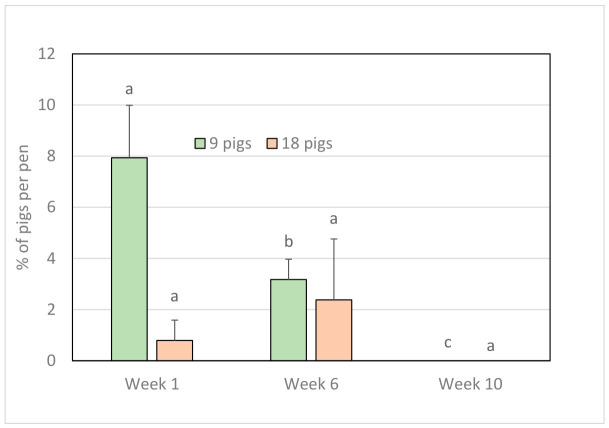
Mean + SE % of pigs per pen with a tucked tail. Tail posture was assessed after the human approach test in Weeks 1, 6 and 10. Different superscrpts (a, b, c) denote significant differences between weeks for group size 9.

The percentage of pigs per pen seeking contact with the assessor in the HAT was higher in pens with 9 pigs than 18 pigs (Table 2). There was an interaction between group size and week, with an increasing percentage of pigs seeking contact over time in groups with 18 pigs but not between Weeks 6 and 10 in groups of 9 pigs (Table 2; Figure 2). In general, the percentage of pigs per pen seeking contact increased over time (Table 3). The percentage of pigs with a tucked tail was not significantly affected by group size per se, but there was a significant interaction between group size and week, showing that the proportion of tucked tails declined significantly over time in the smaller groups, whereas in groups of 18 pigs, this remained similar over time. (Table 2; Figure 3). Similarly, there was a significant interaction between group size and week regarding tail wagging, showing that a lower percentage of pigs were wagging tails in groups of 18 compared to groups of 9 in Week 1 and 10, but not in Week 6 (mean ±SE values: 9 pigs—Week 1: 8.3 ± 2.8, Week 6: 0.0 ± 0.0, Week 10: 2.8 ± 1.8; 18 pigs—Week 1: 4.3 ± 2.1, Week 6: 0.0 ± 0.0, Week 10: 0.0 ± 0.0). More pigs had a curled tail in Weeks 6 and 10 than in Week 1, and fewer had a tucked tail in Week 10 than in previous weeks. In contrast, tail wagging was most pronounced in Week 1 (Table 3). There were no significant effects of feeding system on seeking contact during the HAT or on tail posture (seeking contact: F_1,28_ =0.4, *p* = 0.518; tucked tail: F_1,28_ = 0.4, *p* = 0.559; curled tail: F_1,28_ = 0.2, *p* = 0.676; tail wagging: F_1,28_ = 1.2, *p* = 0.279).

#### 3.1.2. Cleanliness and Locomotion Disorders

There were no effects of group size on the percentage of relatively clean pigs or the percentage with locomotion disorders. Nor were these variables affected by the feeding system (relatively clean pigs: F_1,28_ = 1.5, *p* = 0.239; locomotion disorders: F_1,28_ = 0.0, *p* = 0.994). More pigs were relatively clean in Week 1 than in later weeks (Table 3), but overall, the percentage of relatively clean pigs was high throughout the finishing period. The percentage of pigs with locomotion disorders did not differ significantly per week and was rather low throughout the entire period (Table 3).

#### 3.1.3. Bite Marks

**Figure 4:**
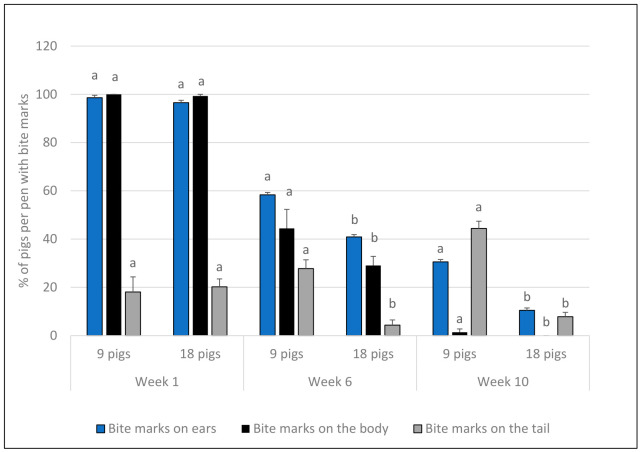
Mean + SE% of pigs per pen with bite marks on ears, tail, and the rest of the body in Week 1, 6 and 10, respectively (mild plus severe combined). Different superscripts (a, b) within same-coloured bars within weeks denote differences between group sizes. The two group sizes differed in all three types of bite marks in Week 6 and 10, while there were no significant differences in any of the bite mark indicator between the group sizes in Week 1. The results show significant differences between group sizes regaring ear and tail bite marks in Weeks 6 and 10 but not in Week 1 (interaction between group size and time).

The percentage of pigs with mild and severe ear bite marks, and in total (mild plus severe), was lower in groups of 18 than 9 pigs (Table 2). The percentages of pigs with severe ear bite marks, and in total, declined over time (Table 3), and the decline in the percentage of pigs with severe bite marks on the ears was more pronounced in the groups of 18 pigs (Table 2; Figure 4). In fact, there were no pigs with severe bite marks on the body or the ears at Week 10. The percentages of pigs with mild bite marks on the body were unaffected by group size (Table 2) but declined over time (Table 3), and the percentage of pigs with severe bite marks on the body showed a greater decline in the groups of 18 pigs (Table 2; Figure 4). The percentage of pigs with mild tail bite marks was lower in groups of 18 pigs than in groups of 9, whereas there was no main group size difference for severe tail bite marks. In groups of 9 pigs, there was an increase in the percentage of pigs with mild tail bite marks with increasing age, whereas there was a decline after Week 1 in groups of 18 pigs (Table 2; interaction effect; Figure 4). The percentage of pigs with mild bite marks on the tail was highest in Week 10, whereas the prevalence of severe tail bite marks was more pronounced in Week 6 (Table 3). There were no significant effects of the feeding system on the proportion of pigs with different types of bite marks (ears in total: F_1,28_ = 0.4, *p* = 0.511; body in total: F_1,28_ = 0.0, *p* = 0.997; tail in total: F_1,28_ = 0.0, *p* = 0.915).

### 3.2. Performance

There were, on average, 2.2 more feeding days before slaughter for pigs kept in groups of 18 than in groups of 9 pigs (Table 4), the feed consumption and feed costs for the farmer but without increasing slaughter weight or lean meat%. None of the other production variables were significantly affected by group size or the interaction between group size and feeding system. Average daily gain, average daily feed intake, and slaughter weight were all higher when pigs were fed ad libitum than when fed restrictively three times a day, whereas the feed conversion ratio did not differ (Table 4).

## 4. Discussion

As predicted, the proportion of pigs with bite marks on the ears and body was lower in pens with the larger group size, though only from Week 6 onwards. At the start of the study, the pigs were mixed with unacquainted individuals when transported to the farm and again when distributed between pens on arrival. Although bite marks on the ears and body were observed throughout the finishing period, the majority of bites, especially severe ones, occurred as a result of fights during mixing with strangers at the start of the study. This explains the substantial decline in ear and body bite mark prevalence from Week 1 (assessed shortly after mixing) to Week 6. The continued decline to Week 10 was presumably based on learned avoidance of specific pigs through keeping at a distance and avoiding visual contact with the head region [21]. It can be speculated (though not supported by the present data) that avoidance would have been easier in larger groups because although the pigs had the same floor space per pig at both group sizes, the total floor space in the pen was larger for the larger group size, allowing the pigs to move farther away and to shelter behind more pigs. In addition, aggressive bites to the ears and body might have been reduced in the larger groups because, throughout the finishing period, the pigs could choose between two feeders, drinkers, resting areas, and enrichment zones instead of just one, which likely reduced competition by reducing the size of aggregations around specific resources. While the location of the slatted area in the centre of the larger pens was unconventional, it contributed to the creation of well-spaced functional areas for the larger groups. It is important to be aware that the results were obtained within this specific farm context, and the pen designs may not be the most optimal.

While the proportion of pigs with tail bite marks declined over time in groups of 18 pigs, it increased over time in groups of 9 pigs. Although the aetiology of tail biting differs from that of social aggression towards strangers, as indicated by higher levels of tail damage in Week 6 (severe) or 10 (mild) than in Week 1, it is likely that providing rooting materials in two different functional areas and the greater ability to avoid specific pigs in larger than smaller groups contributed to the lower levels in the larger groups. Due to the limited amount of litter and rooting material provided, there was not enough space for all individuals to investigate freshly provided rooting material in the smaller pens, especially towards the end of production when the pigs were approaching their maximum weight. This may have created frustration, with some pigs chewing on tails or pen fittings. The timing of tail biting outbreaks varies between studies (e.g., peaking around 4 weeks of age; [22]), and it is difficult to compare studies given differences in animal density, pen design, enrichment strategies, and multiple other factors that may contribute to tail biting [23]. However, the importance of adequate rooting materials for reducing the risk of tail biting was highlighted in a recent study that used a similar pen design and space allocation as those in the current study [24].

The proportion of pigs with tucked tails was lower in the larger groups in Week 1. Along with tail tucking, ear and tail biting are reported to be elevated in sick and uncomfortable pigs [25], suggesting that tail tucking is associated with negative affective states. Our findings are consistent with previous reports that tail tucking is a predictor of tail biting [19,26], confirming that tail posture can be an important welfare indicator [27,28,29]. However, we did not find a higher proportion of pigs with curled or wagging tails in the larger groups. Tail signals are short-lived and vary according to the situation, and it is possible that a single scan for tail postures during the HAT was insufficient to capture group size differences. Curled tails have previously been associated with housing in an enriched vs. barren environment [27], and wagging tails have been associated with locomotion, non-agonistic social interactions [28], playing, and exploring fresh rooting material [18].

Although larger groups are reported to show more locomotion and a higher activity level [14], we did not detect a group size effect on the proportion of pigs with locomotion disorders. The prevalence of locomotion disorders was relatively low in both Weeks 1 and 6 and substantially lower than the levels observed by others [6]. Pig cleanliness did not differ between group sizes, suggesting that removal of the wall across the slatted area to form a larger pen did not adversely impact dunging patterns.

In accordance with recent work [6], pigs in the larger groups were less likely to seek contact with an unfamiliar human in the HAT than those in the smaller groups. Pigs in larger groups may have less contact with humans due to more frequent per-capita human–pig contact in smaller spaces and thus a larger degree of habituation to humans, or they may be more easily able to avoid direct contact with humans due to the larger pen size. However, we could see that more pigs sought human contact with increasing age, especially in the larger groups, such that levels were similar for the two group sizes by Week 10. This is largely due to a growing familiarity with humans treating them in a positive way. According to another field survey [30], farmers who have more confident pigs and better productivity are those who have the farm as their central profession, express the most pleasure in working with pigs and convey empathy for pigs. It should also be mentioned that avoidance of humans by finishing pigs has been related to bite lesions from fighting following mixing [31], suggesting that fear towards humans can also result from factors other than their experiences with humans.

As predicted and in accordance with other work [15], we found that average daily feed intake, average daily gain, and slaughter weight were all higher for the ad libitum treatment than for the feed restriction treatment, even though the amount of feed provided in the latter was adjusted according to body weight and was close to appetite. It is unclear why the expected improvement in feed efficiency for the feed restriction treatment did not occur. However, this could be because the restricted treatment was so close to ad libitum in the amount of food and the adjustment for weight. While performance was better with ad libitum feeding, none of the welfare indicator results differed significantly between the feeding systems, and in contrast to what was predicted, there were no interactions between group size and feeding system. The number of bite marks was expected to be lower in the combination of large groups and ad libitum feeding system, as this should result in a less competitive environment. On the other hand, the “restricted” treatment might not have been restricted enough to produce such effects, at least at the present sample size. This shows that performance and welfare do not necessarily go hand in hand.

## 5. Conclusions

Consistent with the social tolerance hypothesis, the prevalence of bite marks on the ears and tail was lower in groups of 18 pigs than in groups of 9. The percentage of pigs with ear and body bite marks declined over time at both group sizes. However, tail bite marks showed a different pattern: their prevalence increased over time in the smaller groups, whereas it decreased in the larger groups. It is important to note that these results were obtained from an experiment conducted on a single farm within a specific context. With only four replicates per treatment combination, the study is somewhat underpowered to detect moderate effect sizes; therefore, the findings should be interpreted with caution. Apart from pigs in the larger groups requiring approximately 2.2 additional days to reach slaughter weight, group size did not affect performance variables, cleanliness, or the prevalence of locomotion disorders. The fact that pigs in smaller groups reached slaughter weight more quickly resulted in lower feeding costs for the farmer for the smaller groups. Several measures indicated improved welfare when pigs with intact tails were kept in groups of 18 rather than 9, under conditions of partially slatted pens and constant floor space per pig. Ad libitum feeding positively affected performance compared to restricted feeding. Finally, the prevalence of tail bite marks should be considered separately from bite marks on other parts of the body, as tail biting is not primarily associated with aggression. Nevertheless, the effects of group size and time on tail lesions followed patterns similar to those observed for other types of bite marks. Maintaining intact tails is a legal requirement on Norwegian farms, and this study adds to understanding about how tail biting and the resulting lesions are influenced by environmental factors in commercial pig production systems.

## Figures and Tables

**Table 1:** Welfare indicators assessed three times during the finishing period (Week 1, 6, and 10) ^1^.

Welfare Indicator	Category	Description
Response to an unfamiliar human	Seeks contact	Seeks contact with the observer, with the snout less than 10 cm away and attention directed towards the observer.
Tail posture	Tucked	Tail is pressed between the legs.
	Curled	Tail is curled outwards and upwards.
	Wagging	Hanging tail is swaying repeatedly from side to side or tip of curled tail is moving repeatedly in a circular manner.
Pig cleanliness	Relatively clean	≤10% of the body is soiled by manure.
Locomotion disorder	Mild	Slight mobility impairment, stiff gait.
	Severe	Severe mobility impairment, lame, problems with walking and/or standing up from lying.
Bite marks on the ears	Mild	1–4 slightly red or partly healed bite marks on one or both ears.
	Severe	1 or more bleeding bite marks or 5 or more mild bite marks on one or both ears.
Bite marks on the body	Mild	1–4 slightly red or partly healed bite marks.
	Severe	1 or more bleeding bite marks or 5 or more mild bite marks on the body.
Bite marks on the tail	Mild	1–2 slightly red or partly healed bite marks on intact tail or less than a quarter of the tail missing or damaged.
	Severe	1 or more bleeding lesions or quarter or more of the tail missing or damaged.

^1^ To conduct the assessment, an unfamiliar human entered each pen, stood still, and recorded the number of pigs showing contact seeking during a 30 s observation period. This was followed by a scan of the number of pigs with each tail position. The numbers exhibiting each of the remaining indicators were then recorded. Categories within each indicator were mutually exclusive.

**Table 2:** Results of statistical analyses on effects of group size (9 vs. 18 pigs) and interaction of group size with time in the finishing period (Week 1, 6, or 10) on prevalence of each welfare indicator category (% of pigs per group; GLIMMIX model in SAS).

Welfare Indicator Category	Effects of Group Size			Interaction Between Group Size × Time
	9 Pigs	18 Pigs	*p*-Value	*p*-Value
Seeking contact	68.1 ± 3.3	44.4 ± 5.3	<0.0001	0.008
Tucked tail	3.2 ± 1.2	1.2 ± 0.6	0.172	<0.0001
Curled tail	91.7 ± 2.1	88.3 ± 3.9	0.993	0.138
Tail wagging	4.2 ± 1.3	1.4 ± 0.8	<0.0001	<0.0001
Relatively clean	86.1 ± 4.2	91.5 ± 1.9	0.239	0.607
Locomotion disorder (mild + severe)	1.4 ± 0.8	2.1 ± 0.6	0.994	1.0
Mild bite marks on the ears	44.9 ± 4.1	35.8 ± 4.4	0.007	0.021
Severe bite marks on the ears	17.6 ± 4.8	13.5 ± 4.0	0.364	0.0002
All ear bite marks (mild + severe)	62.5 ± 6.3	49.3 ± 7.6	0.020	0.445
Mild bite marks on the body	12.0 ± 3.8	17.8 ± 3.8	0.994	0.886
Severe bite marks on the body	36.6 ± 7.8	24.9 ± 7.0	0.049	<0.0001
All bite marks on the body (mild + severe)	48.6 ± 8.8	42.8 ± 8.8	0.990	0.995
Mild tail bite marks	25.5 ± 3.5	8.6 ± 1.9	<0.0001	0.0001
Severe tail bite marks	4.6 ± 1.5	2.1 ± 0.7	0.264	0.271
All tail bite marks (mild + severe)	30.1 ± 3.4	10.8 ± 2.0	<0.0001	0.0002

**Table 3:** Mean ± SE % of pigs per pen in each welfare indicator category at each assessment time in the finishing period, irrespective of group size and feeding system. Different superscripts (a, b, c) denote significant differences between weeks (*p* < 0.05); GLIMMIX model in SAS; least square means method with Tukey adjustment).

	Time in the Finishing Period			Effect of Time
Welfare Indicator Category	Week 1	Week 6	Week 10	*p*-Value
Seeks contact	37.7 ± 6.3 ^a^	61.2 ± 4.0 ^b^	72.7 ± 3.3 ^c^	<0.0001
Tucked tail	3.8 ± 1.3	2.8 ± 1.5	0.0 ± 0.0	0.949
Curled tail	75.5 ± 4.7 ^a^	95.8 ± 1.7 ^b^	98.6 ± 0.9 ^b^	0.0001
Tail wagging	6.3 ± 1.8 ^a^	0.7 ± 0.7 ^b^	1.4 ± 0.9 ^b^	<0.0001
Relatively clean	98.2 ± 1.4 ^a^	82.9 ± 2.5 ^b^	85.3 ± 5.8 ^b^	0.001
Locomotion disorder (mild + severe)	0.7 ± 0.5	0.4 ± 0.4	4.2 ± 1.1	0.563
Mild bite marks on the ears	55.5 ± 3.3 ^a^	45.1 ± 4.5 ^b^	20.5 ± 3.6 ^c^	<0.0001
Severe bite marks on the ears	42.1 ± 3.4 ^a^	4.5 ± 2.8 ^b^	0.0 ± 0.0 ^c^	<0.0001
All ear bite marks (mild + severe)	97.6 ± 1.5 ^a^	49.6 ± 3.8 ^b^	20.5 ± 3.6 ^c^	<0.0001
Mild bite marks on the body	24.1 ± 6.0	20.0 ± 3.4	0.7 ± 0.7	0.683
Severe bite marks on the body	75.5 ± 6.1 ^a^	16.7 ± 3.9 ^b^	0.0 ± 0.0 ^c^	<0.001
All bite marks on the body (mild + severe)	99.7 ± 0.3 ^a^	36.7 ± 4.7 ^b^	0.7 ± 0.7 ^c^	<0.001
Mild tail bite marks	16.7 ± 3.3 ^a^	10.1 ± 2.5 ^b^	24.4 ± 5.2 ^c^	0.018
Severe tail bite marks	2.5 ± 3.4	5.9 ± 3.4	1.8 ± 3.4	0.271
All tail bite marks (mild + severe)	19.1 ± 3.4 ^a^	16.1 ± 3.7 ^b^	26.1 ± 5.0 ^c^	0.088

**Table 4:** Effects of group size (9 vs. 18 pigs), feeding system (ad libitum vs. restricted), and their interaction on performance of finishing pigs (GLM model in SAS).

	Small Groups *n* = 8 Pens	Large Groups *n* = 8 Pens	Group Size	Ad libitum	Restricted	Feeding System Effect	Group Size × Feeding System Effect
	mean ± SE	mean ± SE	*p*-value	mean ± SE	mean ± SE	*p*-value	*p*-value
Body weight at start (kg)	33.4 ± 0.55	33.0 ± 0.78	0.66	33.6 ± 0.5	33.0 ± 0.8	0.45	0.86
Average daily gain (g)	1222 ± 11.8	1212 ± 16.8	0.49	1251.5 ± 10.6	1185 ± 8.3	<0.001	0.94
Average daily feed intake (kg)	2.9 ± 0.03	2.9 ± 0.04	0.68	3.0 ± 0.0	2.7 ± 0.0	0.002	0.85
Feed conversion ratio (kg feed/kg slaughter weight)	2.41 ± 0.018	2.42 ± 0.025	0.84	2.7 ± 0.0	2.7 ± 0.0	0.71	0.88
Days to slaughter	86.2 ± 0.55	88.6 ± 0.78	0.012	85.9 ± 0.9	88.1 ± 0.6	0.085	0.29
Slaughter weight (kg)	94.2 ± 0.65	95.6 ± 0.92	0.21	95.9 ± 0.7	93.5 ± 0.7	0.030	0.46
Lean meat (%)	59.2 ± 0.23	59.7 ± 0.33	0.18	59.4 ± 0.2	59.4 ± 0.3	0.22	0.20

## Data Availability

The datasets generated and/or analysed during the study are available from the corresponding author upon request.
